# Paraneoplastic CDR2 and CDR2L antibodies affect Purkinje cell calcium homeostasis

**DOI:** 10.1007/s00401-014-1351-6

**Published:** 2014-10-24

**Authors:** Manja Schubert, Debabrata Panja, Mette Haugen, Clive R. Bramham, Christian A. Vedeler

**Affiliations:** 1 Department of Neurology, Haukeland University Hospital, 5021 Bergen, Norway; 2 Department of Clinical Medicine (K1), University of Bergen, 5021 Bergen, Norway; 3 Department of Biomedicine and KG Jebsen Centre for Research on Neuropsychiatric Disorders, University of Bergen, 5009 Bergen, Norway

**Keywords:** Calbindin D_28K_, Calcium homeostasis, Paraneoplastic cerebellar degeneration, Onconeural Yo antibodies, Purkinje cell death, Purkinje cell-specific protein-2

## Abstract

Paraneoplastic cerebellar degeneration (PCD) is characterized by loss of Purkinje cells (PCs) associated with progressive pancerebellar dysfunction in the presence of onconeural Yo antibodies. These antibodies recognize the cerebellar degeneration-related antigens CDR2 and CDR2L. Response to PCD therapy is disappointing due to limited understanding of the neuropathological mechanisms. Here, we report the pathological role of CDR antibodies on the calcium homeostasis in PCs. We developed an antibody-mediated PCD model based on co-incubation of cerebellar organotypic slice culture with human patient serum or rabbit CDR2 and CDR2L antibodies. The CDR antibody-induced pathology was investigated by high-resolution multiphoton imaging and biochemical analysis. Both human and rabbit CDR antibodies were rapidly internalized by PCs and led to reduced immunoreactivity of calbindin D_28K_ (CB) and L7/Pcp-2 as well as reduced dendritic arborizations in the remaining PCs. Washout of the CDR antibodies partially recovered CB immunoreactivity, suggesting a transient structural change in CB calcium-binding site. We discovered that CDR2 and CB co-immunoprecipitate. Furthermore, the expression levels of voltage-gated calcium channel Cav2.1, protein kinase C gamma and calcium-dependent protease, calpain-2, were increased after CDR antibody internalization. Inhibition of these signaling pathways prevented or attenuated CDR antibody-induced CB and L7/Pcp-2 immunoreactivity loss, morphological changes and increased protein expression. These results signify that CDR antibody internalization causes dysregulation of cell calcium homeostasis. Hence, drugs that modulate these events may represent novel neuroprotective therapies that limit the damaging effects of CDR antibodies and prevent PC neurodegeneration.

**Electronic supplementary material:**

The online version of this article (doi:10.1007/s00401-014-1351-6) contains supplementary material, which is available to authorized users.

## Introduction

Paraneoplastic cerebellar degeneration (PCD), caused by Purkinje cell (PC) death in the cerebellum, is a paraneoplastic neurological disorder with severe pancerebellar symptoms, such as ataxia, nystagmus, and dysarthria [72]. Cross-reactivity of onconeuronal Yo antibodies (Yo Abs) and T cells with antigens in the tumor tissue and cerebellum induce PCD [63]. Yo Abs bind to the cytoplasmic antigen of cerebellar degeneration-related protein 2 (CDR2, 50 kDa, RefSeq NM_001802.1) and 2-like (CDR2L, 62 kDa, RefSeq NM_014603.2) and are found in the serum and cerebrospinal fluid of patients with remote, non-metastatic ovarian and breast cancers [7, 22, 72].

Brain, normal ovary tissue, and tumor tissue express CDR2 protein [15, 30, 70]. CDR2 interacts with proteins involved in signal transduction and gene transcription such as: cell cycle-related proteins; PKN, a fatty acid-activated serine/threonine protein kinase; and c-myc [48, 49, 54, 55, 66]. CDR2L has ~50 % sequence identity with CDR2, but is not expressed in PCD tumor tissue [15]. In the cerebellum, both CDR2 and CDR2L proteins are present in the cytoplasm and proximal dendrites of PCs, but their functions are unknown [15, 49, 52].

The PCs are the sole projection neurons in the cerebellum and therefore proper morphological as well as physiological integrity of the PC dendrites is essential for cerebellar function [25, 67]. Optimal intracellular Ca^2+^ levels and Ca^2+^ flux via cytoplasmic Ca^2+^-binding protein calbindin D_28K_ (CB) and the GoLoco domain protein, Purkinje cell-specific protein-2 (L7/Pcp-2), are essential for cellular and molecular mechanisms involved in neurotransmitter release, ion channel permeability, enzyme activity, and gene transcription [6, 24, 39, 57]. With its four Ca^2+^-binding sites, CB is a Ca^2+^ buffer and sensor; it regulates fast Ca^2+^ influx by Ca^2+^ binding [57] and is considered as a PC survival marker [28, 38]. L7/Pcp-2 modulates P/Q-type voltage-gated calcium channels’ (VGCC) function, whose dysfunction is implicated in ataxia [27, 39, 69]. Factors that modulate or disrupt CB and L7/Pcp-2 are expected to exert powerful physiological and pathological effects on PCs and are listed in Table S1.

In the present study, an ex vivo model of rat cerebellar organotypic slice culture (cOTSC) was used to study the neuropathological mechanisms underlying the antibody-mediated PCD and to identify potential intracellular treatment targets which are elaborated in Table S1. Multiphoton imaging showed that CDR2 and CDR2L antibody internalization reduced the CB and L7/Pcp-2 immunoreactivity levels in PCs. This antibody-driven immunoreactivity loss was reduced by modifying the intracellular Ca^2+^ transients and inhibiting the Ca^2+^-dependent protease calpain (Table 1). These findings suggest that widespread consequences of Ca^2+^ homeostasis dysregulation can induce morphological changes and fatal alterations in the cell signaling pathways, thereby causing neurodegeneration.

**Table 1:** Percentage loss of CB^+^- and L7/Pcp-2^+^-PCs after CDR antibody internalization including additional drug treatment in comparison to control (non-*h*CDR or *r*IgG)

Duration (days)	Treatment	Loss of CB^+^ PCs							Loss of L7/Pcp-2^+^ PCs				
		*h*CDR2^(PS1)^	*h*CDR2L^(PS2)^	*h*CDR2/2L^(PS3)^	*h*CDR2/2L^(PS4)^	*r*CDR2	*r*CDR2L	*r*CDR2/2L	*h*CDR2/2L^(PS3)^	*h*CDR2/2L^(PS4)^	*r*CDR2	*r*CDR2L	*r*CDR2/2L
2	CDR-Abs	−11 ± 7 %	−33 ± 9 %	−22 ± 7 %	−56 ± 7 %	−47 ± 7 %	−56 ± 4 %	−58 ± 6 %	ND	−58 ± 8 %	ND	ND	ND
4	CDR-Abs	−52 ± 8 %	−55 ± 9 %	−57 ± 11 %	−73 ± 6 %	−72 ± 9 %	−69 ± 8 %	−77 ± 6 %	ND	−60 ± 8 %	ND	ND	ND
6	CDR-Abs	−71 ± 12 %	−80 ± 11 %	−79 ± 9 %	−77 ± 8 %	−79 ± 13 %	−76 ± 10 %	−85 ± 8 %	−65 ± 10 %	−74 ± 8 %	−69 ± 3 %	−66 ± 2 %	−74 ± 12 %
7	CDR-Abs WASHOUT	ND	ND	ND	ND	−40 ± 10 %	−33 ± 8 %	−43 ± 9 %	ND	ND	ND	ND	ND
6	CDR-Abs + block VGCC (150 nM agatoxin)	ND	ND	ND	ND	+6 ± 9 %^a^	−5 ± 11 %	+15 ± 13 %^a^	ND	ND	+5 ± 9 %^a^	+15 ± 13 %^a^	+31 ± 15 %^a^
6	CDR-Abs + block AMPAR (10 μM CNQX)	ND	ND	−24 ± 8 %	−34 ± 6 %	−41 ± 4 %	−24 ± 5 %	ND	ND	−38 ± 5 %	−48 ± 4 %	−27 ± 5 %	ND
6	CDR-Abs + block PKC (50 nM K252a)	ND	ND	−8 ± 7 %	ND	−11 ± 4 %	−6 ± 6 %	ND	−10 ± 6 %	ND	−12 ± 4 %	−6 ± 6 %	ND
6	CDR-Abs + block calpain (10 μM MDL28170)	ND	ND	−42 ± 9 %	ND	−21 ± 10 %	−43 ± 12 %	−33 ± 12 %	ND	−49 ± 5 %	ND	ND	ND

*Abs* antibodies, *ND* not determined, *PS* patient serum

^a^Increased cell count compared to control; values of mean ± SEM

## Materials and methods

### Patients’ sera

We used four female patients’ sera that were antibody positive for CDR2 (*h*CDR2^+(PS1)^), CDR2L (*h*CDR2L^+(PS2)^) or both CDR2 and CDR2L (*h*CDR2/2L^+(PS3/PS4)^) [22] and negative for P/Q-type VGCC (RIA; DLD Diagnostika, #RA006/12). Patient data are listed in Table S2. The sera were collected before treatment took place and stored at −80 °C at the Paraneoplastic Neurological Diseases Bio-bank (#484) with the approval of the Regional Committee for Medical and Health Research Ethics in Western Norway, Diagnostic markers of cancer (188.05). As control serum, we pooled samples from 100 healthy blood donors without any known autoimmune disease (non-*h*CDR).

### Cerebellar organotypic slice culture (cOTSC)

**Fig. 1:**
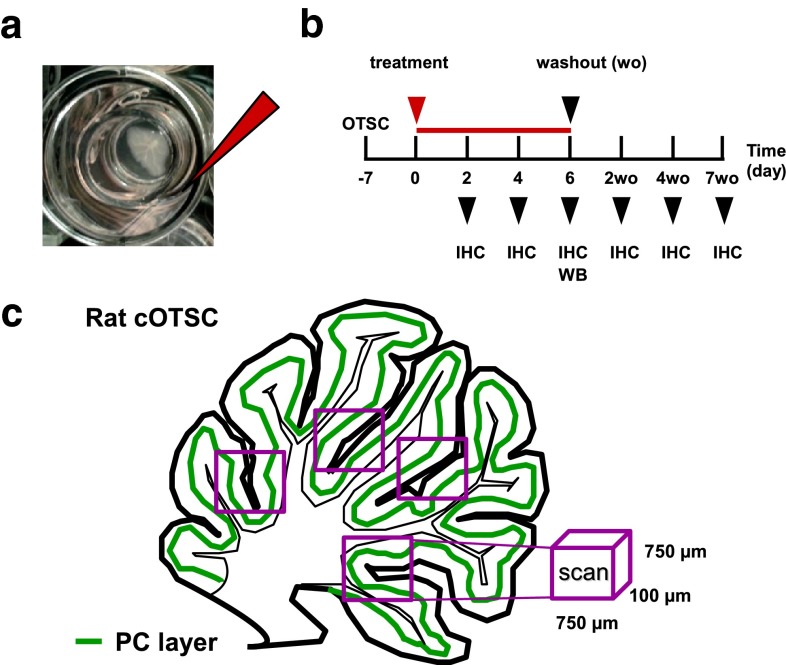
CDR antibody-mediated PCD model. **a** Ex-vivo PCD model system of cerebellar organotypic slice culture (cOTSC). **b** Experimental design used to investigate the CDR antibody pathology on cerebellar PC physiology. *h/r*CDR were added to the culture media of cOTSC 7 days after preparation (treatment, *red arrow*). Pathological effects of the administered treatment were analyzed 2, 4, and 6 days later by immunohistochemistry (IHC) and Western blot analysis (WB). The reversibility of the observed CDR-induced pathology was tested by IHC at 2, 4, and 7 days after 6 days of CDR treatment (washout). **c** Multiphoton micrographs were collected, and PC were counted on three to eight scans of 0.056 mm³ for each treated cOTSC slice to determine the effects of CDR treatment

All procedures were performed according to the National Institutes of Health Guidelines for the Care and Use of Laboratory Animals Norway (FOTS 20135149/20113133). To prepare cOTSC, we used 152 Wistar Hannover GLAST rat pups (in-house breeding colony), age P10–P15. Following anesthesia and decapitation, we transferred the cerebellum into ice-cold EBSS solution (Gibco, #24010043) containing 0.5 % D-glucose (Sigma, #G8769) and 10 mM HEPES (Gibco, #15630056). Four to five cerebellar parasagittal slices (400 μm thick) were cut on NVSLM1 motorized advance vibroslice (WPI) and transferred individually onto 0.4 μm pore size membranes (Millicell, Millipore, # PICMO1250). Slices were maintained in 24-well plates at the air/culture media interface consisting of 30 % advanced DMEM/F12 solution (Gibco, #126340010), 20 % MEM solution (Gibco, #41090028), 25 % EBSS solution, 25 % heat-inactivated horse serum (Sigma, #H1138), 1 mM l-glutamine (Gibco, #35050038), 5 mg/mL d-glucose, and 2 % B-27 serum-free supplement (Gibco, #17504044), and incubated with 5 % CO_2_ at 35 °C (Fig. 1a). The culture medium was removed and replaced 24 h post-slicing (100 %) and then every 2nd day (75 %). The slices recovered for 7 days before treatment.

### Antibody-mediated PCD model: patients’ sera (*h*CDR) and polyclonal affinity-purified rabbit antibodies (*r*CDR)

*h*CDR and *r*CDR were heat inactivated (56 °C, 30 min) to destroy complement factors prior to treatment to obtain neutral IgG antibody assays and added at various concentrations to the cOTSC media for 2–6 days (Fig. 1b). We used human sera *h*CDR2^+^, *h*CDR2L^+^, and *h*CDR2/2L^+^, non-*h*CDR at dilutions of 1:250, 1:500, and 1:1000, and affinity-purified polyclonal rabbit antibody *r*CDR2 (Sigma, #HPA023870), *r*CDR2L (Sigma, #HPA022015), or both (1:1 mixture) and *r*IgG (Millipore, #12370) in total concentrations of 20, 40, 125, or 400 ng/mL. Each independent experiment (*n* ≥ 3) included positive and negative controls to avoid variations in immunoreactivity to the applied *h*CDR and *r*CDR between each experiment.

### Neuropharmacology

The following drugs were used: *MDL28170* a potent, selective inhibitor of calpain (*K*_i_ = 10 nM; Tocris, #1146); *CNQX* an AMPA receptor antagonist (IC_50_ = 1–2 μM; Tocris, #1045); *ω*-*agatoxin* a selective and reversible blocker of Cav2.1 (P/Q-type VGCC) (Alomone, #A-530); *K252a* a non-selective protein kinase inhibitor that inhibits PKC (IC_50_ = 32.9 nM; Tocris, #1683); *U0126* a selective non-competitive inhibitor of MEK-1 and MEK-2 (IC_50_ = 60–70 nM; Tocris, #1144).

### Primary antibodies

The antibodies used in immunohistochemical (IHC), Western blot (WB), and immunoprecipitation (IP) analyses are detailed in Table S3.

### Immunohistochemistry

#### cOTSC sections

After treatment, cOTSC were washed with pre-warmed 0.1 M PBS (1xPBS; Gibco, #70013016) and fixed (4 % paraformaldehyde (PFA)/0.5 % sucrose in PBS, pH 7.2; 4 h, 4 °C). Slices were quenched with PBS/50 mM NH_4_Cl (PBS_N_), permeabilized with PBS_N_/1 % Triton X-100 (60 min, 22 °C), rinsed (3 × 5 min) with PBS_N_, and incubated in primary Ab against calbindin D_28K_, caspase-3, or L7/Pcp-2 for 2 days at 4 °C in PBS_N_ containing 5 % bovine serum albumin (BSA; Sigma, #A2153), 0.2 % Triton X-100 (Sigma, #T9284), and 100 μM glycine (Sigma, #G7126). The slices were washed (3 × 5 min) with PBS_N_ and incubated with 2nd Ab (Alexa Fluor^®^ 488/594 Donkey Anti-Mouse and/or Donkey Anti-Rabbit IgG (H + L), 1:500; Molecular Probes, #A21202, #A21203, #A21204, or # A21207) for 2 days at 4 °C in PBS_N_/2.5 % BSA. Slices were rinsed (3 × 5 min) with PBS_N_ and mounted with PromoFluor Antifade Reagent (Promokine, #PK-PF-AFR1). The slices from each experiment were stained simultaneously to minimize variations in immunoreactivity of primary and 2nd Ab solution within the investigated groups.

#### Cryostat sections

Anesthetized adult female rats were transcardially perfused with ice-cold 4 % PFA–PBS. The brains were post-fixed (24 h, 4 °C), incubated in 18 % sucrose–PBS (72 h, 4 °C), snap-frozen, and cut on a cryostat into 8 μm parasagittal sections. Sections were air dried (30 min, 22 °C), blocked in PBS/0.2 % BSA/1 % Triton X-100 (PBS_B_, 2 h, 22 °C), incubated in patient serum (PBS_B_/patient serum, overnight, 4 °C, 1:2000), rinsed (3 × 5 min) with PBS, incubated with 2nd Ab (PBS_B_/Alexa Fluor^®^ 488 Goat Anti-Human IgG (H + L), 1:500, # A11013, Molecular Probes, 2 h, 22 °C), rinsed (3 × 5 min) with PBS, and mounted with ProlongGold Antifade Reagent (Invitrogen, #P36931). Slices were scanned with a DM6000 CFS-TCS SP5 confocal microscope (Leica).

#### Paraffin-embedded sections

Six days after *h*CDR2/2L and non-*h*CDR internalization, slices were fixed (4 % PFA), embedded in paraffin, sliced into 4 µm thick sections, and stained with hematoxylin and eosin (HE). Images of the HE stain sections were taken with a 40 × 0.65 air objective on Leica DMLS with AxioCam MRC (Zenlite 2011, Zeis) at 100 ms exposure time.

### Multiphoton imaging

**Fig. 2:**
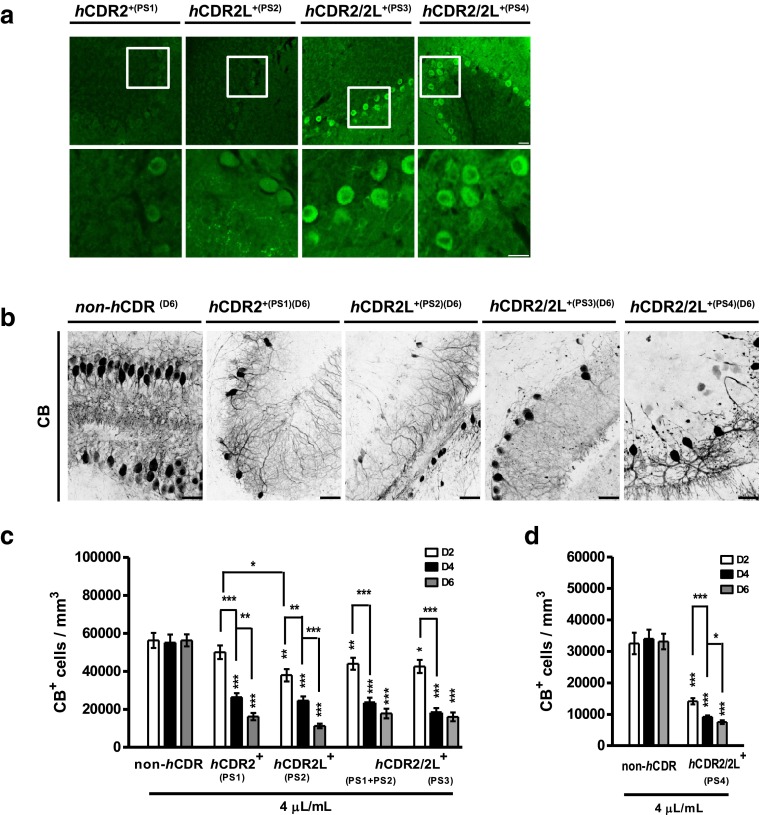
Calbindin D_28K_ immunoreactivity loss in Purkinje cells in response to experimental induced human CDR antibody-mediated PCD. **a** Human CDR (*h*CDR)/PC binding was visualized by anti-human IgG AF488 staining of 8 μm cryostat rat cerebellum sections incubated with *h*CDR^+^ patients’ sera (1:2000). Magnification of the conducted cerebellar PC region micrographs (*lower panel*) shows that both *h*CDR2 and *h*CDR2L antibodies bound to the PC soma. *Scale bars* 25 μm. **b** Multiphoton micrographs of cOTSCs: Independent of the Ab target, all *h*CDR sera (4 μL/mL; 6 days; PS1-4) led to CB-positive (CB^+^) PC loss and altered their dentritic morphology; *scale bars* 40 μm. **c**, **d** Calculation of CB^+^ cells/mm^3^ after 2, 4, and 6 days of hCDR internalization revealed pathological effects of *h*CDR compared to non-*h*CDR control over time (n_E_ = 4). Data in mean ± SEM. Non-parametric two-tailed paired Mann–Whitney’s *U* test. *p < 0.05; **p < 0.01; ***p < 0.001. The percentage changes to controls are summarized in Table 1

**Fig. 3:**
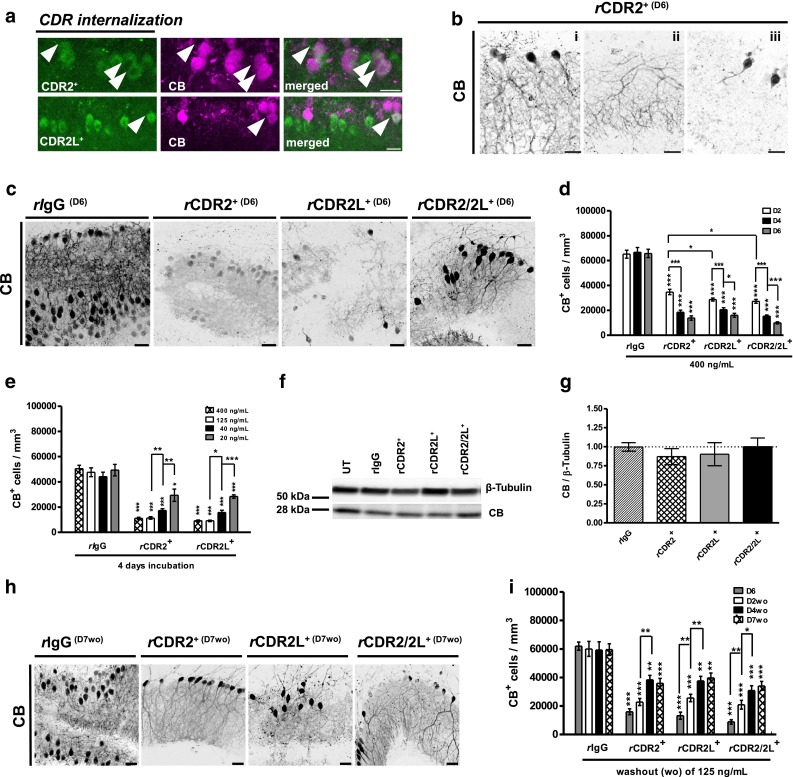
Affinity-purified rabbit CDR-Abs (*r*CDR) reduce calbindin D_28K_ immunoreactivity in a time- and concentration-dependent manner with partial recovery after CDR-Ab washout. **a** *r*CDR2 and *r*CDR2L (400 ng/mL, 6D) are independently internalized into PCs (*green*). Only few CDR-marked cells (*arrow*) are positively correlated with calbindin D_28K_ (CB, *magenta*); *scale bars* 20 μm. We found different expression patterns of CB immunoreactivity and PC morphology modifications for *r*CDR2 **b**, *r*CDR2L, and *r*CDR2/2L compared to *r*IgG control (**c**); *scale bars* 40 μm. The CB immunoreactivity loss, studied 2, 4, and 6 days of *r*CDR internalization by stereological counting of CB^+^ cells/mm^3^, was time- (**d**) and concentration- (**e**) dependent (time: *n*_E_ = 4; concentration: *n*_E_ = 3). **f**, **g** Western blot analysis of *r*CDR-treated cOTSC lysate (125 ng/mL, D6) showed no difference in CB protein levels to *r*IgG controls (*n*_E_ = 10). **h** CB^+^-PC immunoreactivity loss at day 6 of *r*CDR internalization was partially rescued 7 days (D7wo) post-*r*CDR washout (*scale bars* 40 μm), but did not reach control level and plateaued between washout days 4 and 7 (*n*_E_ = 3) (**i**). Data in mean ± SEM. Non-parametric two-tailed paired Mann–Whitney’s *U* test. *p < 0.05; **p < 0.01; ***p < 0.001. Table 1: CDR-Ab effects in percentage

Multiphoton images were collected with a Ti Sapp laser (Coherent Chameleon Ultra2) and DM6000 CFS-TCS SP5 microscope (Leica) using an HCX PL APO 20 × 1.0 water-immersion objective (with a digital zoom of ×1.7 or ×3.0 [Fig. 3b]). Excitation was performed at 740 nm (6.8–7.2 mW laser power); emission was detected for Alexa Fluor^®^ 488/594 with the NDD1/NDD2 external detectors, respectively. The fluorescence intensity was adjusted to 75 % of the maximum in untreated controls for each experiment. Z-stack images were taken at 0.5–1 μm intervals. Pictures were superimposed using LASAF software version 2.5.1 (Leica Microsystems CMS GmbH). Additionally, images from Alexa Fluor^®^ 488/594 Donkey Anti-Mouse IgG staining in Figs. 2b, 3b, c, h were pseudo-colored in gray and inverted for clarity using Fiji. Figure S1b was created as 3D projections with orthogonal section from z-stacks using the Fiji plug-in 3D Viewer. Calbindin D_28K_ (CB^+^)- and L7/Pcp-2 (L7/Pcp-2^+^)-positive PCs were counted manually and automatically, but blind in three to eight images of 750 × 750 × 100 μm in each slice for each experiment (*n*_E_) and group and projected to mm^3^ (Fig. 1c). The automatic count was performed with Fiji: (1) image → stack → plug-in: z projection [max intensity]; (2.) image → adjustment → plug-in: auto threshold [yen]; (3) analyze → plug-in: 3D objects counter [threshold: 50; size filter: min: 200 and max: 1000]. The manual and automatic counts produced equivalent numbers.

### Western blot analysis

The treated cOTSC were washed with pre-warmed 1xPBS (pH 7.4) and sonicated in ice-cold lysis buffer (in mM: 50 Tris–HCl (pH 7.5), 150 KCl, 1 DTT, 1 PMFS, 0.1 % NP40, 1 % SDS, and complete protease inhibitor cocktail (Roche, #1187380001)). The lysates (40 μg) were subjected to gradient SDS-PAGE under reducing conditions (15–4 % Tris TGX gels; Bio-Rad, #456-1086), transferred onto PVDF membrane (0.2 μm pore size; Bio-Rad, #162-0177) and incubated overnight (4 °C) with anti-calbindin D_28K_, anti-calpain-1, anti-calpain-2, anti-Cav2.1, anti-PKCγ, and anti-β-tubulin. The blots were developed using horseradish peroxidase-conjugated 2nd Ab (polyclonal Swine Anti-Rabbit IgG-HRP, Dako, #P0217; Goat Anti-Mouse IgG-HRP, Santa Cruz Biotechnology Inc., #SC-2500; 90 min, 22 °C) and the Pierce ECL chemiluminescence system (Thermo Fisher Scientific Inc., #3210634075). Semi-quantitative densitometry analysis was performed (Bio-Rad, Quantity One 4.6.6). For each sample, the protein level was normalized to β-tubulin and expressed relative to untreated naive control, set at 1 (arbitrary units).

### Protein complex immunoprecipitation (Co-IP)

Co-IP was performed with rat cerebellar tissue. The tissue was homogenized by 10–15 strokes in a Dounce-type glass homogenizer (buffer in mM: 20 Tris–HCl (pH 7.4), 137 NaCl, 10 % glycerol, 1 EDTA, 1 PMSF, 1 DTT, 1 % NP-40, protease inhibitors). Homogenates were centrifuged (13,000*g*, 4 °C, 10 min) and the supernatants were used for immunoprecipitation after pre-clearing with protein G magnetic beads (1 h, 4 °C; Millipore, # LSKMAGG02). The supernatants were incubated with 2 μg/mL of specific Ab with constant agitation (overnight, 4 °C). A further 40 μL of fresh protein G magnetic beads was added and incubated for 1 h at 4 °C. The beads were washed three times (IP buffer in mM: 50 Tris–HCl (pH 7.4), 150 NaCl, 1 EDTA, 1 PMSF, 1 DTT, 0.1 % NP-40, protease inhibitors) and incubated with 100 mM dithiothreitol and 2× Laemmli sample buffer (5 min, 95 °C). Eluted proteins were resolved by SDS-PAGE. We immunoprecipitated against: anti-CDR2, anti-CDR2L, anti-Cav2.1, and anti-calbindin D_28k_ (2 μg).

### Data analysis and statistics

Data analysis and calculations were performed using the software Excel 2003 and Graph Pad Prism 4.0. Data are presented as mean ± SEM, and statistical significance was determined using the non-parametric two-tailed paired Mann–Whitney’s *U* test. The level of significance is indicated with asterisks: *p < 0.05; **p < 0.01; ***p < 0.001.

## Results

### Calbindin D_28K_ (CB) function is modified by *h*CDR and *r*CDR antibodies

We investigated four female patients’ sera that were positive for CDR2, CDR2L, or both CDR2/2L and negative for P/Q-type VGCC (Table S2). Cerebellum cryostat sections incubated with those human patients’ sera (*h*CDR; 1:2000) confirmed that *h*CDRs bound to PCs. *h*CDR2/2L^+(PS3/PS4)^ showed stronger PC binding than *h*CDR2^+(PS1)^ and *h*CDR2L^+(PS2)^ (Fig. 2a).

To evaluate the pathological effects of *h*CDR internalization into PCs in the absence of the lymphatic system and blood–brain barrier, we applied the sera to cOTSC (Fig. 1a). We collected samples at different time points and studied PC survival by counting CB-positive PCs in multiphoton micrographs (Fig. 1b, c) [28, 38]. To exclude Ab diffusion into dead PCs with permeable membranes, we performed control monoclonal anti-CB staining without Triton X-100 in the incubation buffers. We did not find false-positive CB immunoreactivity (data not shown).

*h*CDR internalization significantly reduced the number of CB-positive PCs (CB^+^-PCs) over time (*n*_E_ = 4; Fig. 2c, d; Table 1). The remaining CB^+^-PCs showed reduced dendritic arborizations (Fig. 2b). Within 48 h, CB^+^-PC loss was significantly higher in *h*CDR2L^+(PS2)^ than *h*CDR2^+(PS1)^ (*p = 0.0373; Fig. 2c), but did not differ between *h*CDR2/2L^+(PS3)^ and *h*CDR2/2L^+(PS1+PS2)^ and was most strongly pronounced in *h*CDR2/2L^+(PS4)^ (Fig. 2d; Table 1). Compared to the non-*h*CDR control, more than 70 % of CB^+^-PCs were lost at day 6 of *h*CDR internalization (***p < 0.0001; Fig. 2c, d; Table 1).

To elucidate whether the observed pathology was induced by CDR antibodies, we treated the cOTSC with heat-inactivated affinity-purified polyclonal rabbit Abs against CDR2 (*r*CDR2^+^) and CDR2L (*r*CDR2L^+^), or both (1:1 mixture, *r*CDR2/2L). We found that within 6 days both *r*CDR2 and *r*CDR2L were internalized and led to extensive loss of CB staining (400 ng/mL; Fig. 3a, merged). We observed three patterns of CB immunoreactivity modifications after *r*CDR treatment: (1) whole cell staining with neighboring cells unstained, (2) dendritic staining with only a weak remaining signal in the soma, and (3) soma staining with no dendritic staining (Fig. 3b). The remaining CB^+^-PCs showed morphological alterations in their dendritic arborizations with a loss of tertiary branches (Fig. 3c).

To verify the progression of *r*CDR-induced PCD-like pathology, samples were collected at different time points (*n*_E_ = 4; 400 ng/mL; Fig. 3c, d). We found no effect on the number of CB^+^-PCs in *r*IgG control. Meanwhile within 48 h, we observed that *r*CDR treatment caused a 50–60 % CB^+^-PCs loss. The CB^+^-PC loss under *r*CDR2/2L and *r*CDR2L was of similar magnitude, but significantly greater than in *r*CDR2 (*n*_E_ = 4, *p = 0.0369; Fig. 3d; Table 1). *r*CDR reduced CB^+^-PCs by ~80 % within 6 days (*r*CDR groups compared to *r*IgG^+^ ***p < 0.0001; Fig. 3d; Table 1). The progression of the antibody-driven loss of CB in the first 48 h was similar in *r*CDR2 and *r*CDR2L compared to *h*CDR2^+(PS1)^ and *h*CDR2L^+(PS2)^ (Table 1).

To study antibody level effects, we tested four *r*CDR concentrations (ng/mL): 400, 125, 40, and 20 (*n*_E_ = 3, 4 days). We found a reduction of CB^+^-PCs by ~80 % (400 and 125 ng/mL), 60 % (40 ng/mL), and 40 % (20 ng/mL) compared to *r*IgG control (***p < 0.0001; Fig. 3e) with no difference between *r*CDR2 and *r*CDR2L.

The Western blot analysis of *r*CDR-treated cOTSC lysates (6 days, 125 ng/mL) showed no effect on CB protein concentration compared to untreated naive control (*n*_E_ = 10; Fig. 3f, g). It is therefore likely that the reduced CB immunoreactivity is caused by structural modifications of the CB Ca^2+^-binding site or misfolding of the protein itself [11, 50]. To investigate this hypothesis, we replaced the *r*CDR-containing media (125 ng/mL, 6 days) with non-CDR media (Fig. 1b, washout, 7 days). The number of CB^+^-PCs in *r*IgG control was not affected by washout or by time (Fig. 3h, i). We found that the washout of *r*CDR improved the CB^+^-PC count in *r*CDR2L and *r*CDR2/2L -treated cOTSC within 48 h (D2wo) (*r*CDR2: *p* = 0.1383, *r*CDR2L: **p = 0.0018, *r*CDR2/2L: **p = 0.0010; Fig. 3i). All *r*CDR groups showed substantial recovery of CB^+^-PCs at day 7 after washout (***p < 0.0001; Fig. 3h, i; Table 1), but plateaued between days 4 and 7 at ~ 40 % CB^+^-PC loss before reaching the *r*IgG control count. This supports our hypothesis that the CB Ca^2+^-binding epitope is reversibly altered or blocked, or that the protein might be misfolded.

**Fig. 4:**
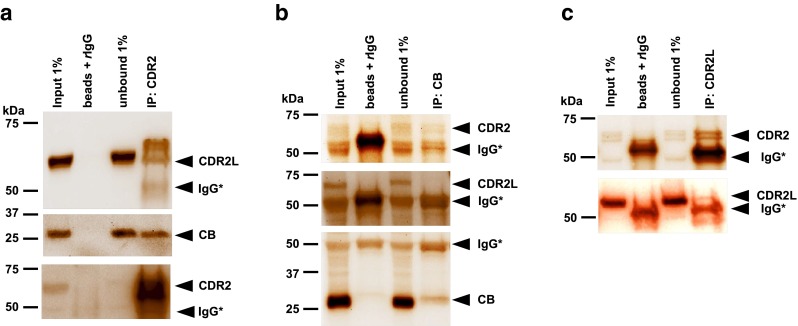
CB Co-IPed with CDR2 but not with CDR2L. **a**, **b**, **c** cerebellar lysates were subjected to co-immunoprecipitation (co-IP) with anti-CDR2, -CDR2L, or -CB Abs, and the precipitated proteins (IP) were immunoblotted with CDR2, CDR2L, and CB (Co-IP). The blots consist of four lanes: input (1 % rat cerebellar lysates), beads + *r*IgG (lysates with 2 μg of species-specific IgG), unbound (1 % supernatant after IP with specific antibody) and IP (Protein complexes recovered after Co-IP with specific antibody as indicated in the figure). *Arrowheads*: probed protein and nonspecific IgG* (heavy chain, 55 kDa). *Left*: molecular mass markers (kDa). Western blots of proteins recovered after IP of the CDR2 complex **a** showed that CB and CDR2L interact with CDR2, which was endorsed by reciprocally Co-IP (**b**, **c**). However, CDR2L was not Co-IPed with CB (**b**)

To analyze whether CDR proteins and CB interact at physiological expression levels, we assayed by reciprocal co-immunoprecipitation (Co-IP) CDR2, CDR2L, and CB proteins from cerebellar lysates. As shown in Fig. 4a, b, CB and CDR2 specifically Co-IPed, indicating endogenous CDR2–CB complex in PCs. Interestingly, CDR2L did not form any complex with CB (anti-CDR2L from three sources), but showed strong CDR2 Co-IP (Fig. 4a, c). These Co-IP results suggest that CDR2 antibody can substantially influence CB function by binding to an interacting protein.

Although CB immunoreactivity was reduced after *h*CDR and *r*CDR internalization, the micrographs in Fig. 3a showed no cell loss. HE staining of *h*CDR2/2L^+(PS3)^-treated cOTSC (1 μL/mL, 6 days) showed normal cytoarchitecture and no distinct loss of PCs (Fig. S1a). Furthermore, staining for the apoptotic marker of active cleaved caspase-3 in *r*CDR2/2L-treated cOTSC (100 ng/mL, 6 days) showed small scattered cleaved caspase-3-positive apoptotic cells which did not co-localize with CB^+^-PCs (Fig. S1b). This caspase-3 staining profile was not different from the *r*IgG control.

### GoLoco domain protein L7/Pcp-2 is affected by *h*CDR and *r*CDR antibodies

PCs express GoLoco domain protein L7/Pcp-2, which interacts with Gα_o_ and Gα_i_ proteins and thereby modulate the P/Q-type VGCC (Cav2.1) functions in a concentration-dependent manner [39]. The P/Q-type is the major VGCC in PCs and linked to ataxia pathogenesis [27, 69]. To evaluate whether L7/Pcp-2 is influenced by CDR internalization, we stained *h*CDR- and *r*CDR-treated cOTSC against L7/Pcp-2. The L7/Pcp-2 positive (L7/Pcp-2^+^) PC loss was similar to the observed CB^+^-PC loss (Fig. S2; Table 1). Both *h*CDR (1 μL/mL) and *r*CDR (100 ng/mL) induced L7/Pcp-2^+^-PC loss of more than 65 % (6 days, *h*CDR *n*_E_ = 3; *r*CDR *n*_E_ = 4; compared to *r*IgG control ***p < 0.0001; Fig. S2a–d; Table 1).

This suggests that another important Ca^2+^ homeostasis regulator is affected by CDR antibody internalization.

### VGCC Cav2.1 is up-regulated by *h*CDR and *r*CDR antibodies

**Fig. 5:**
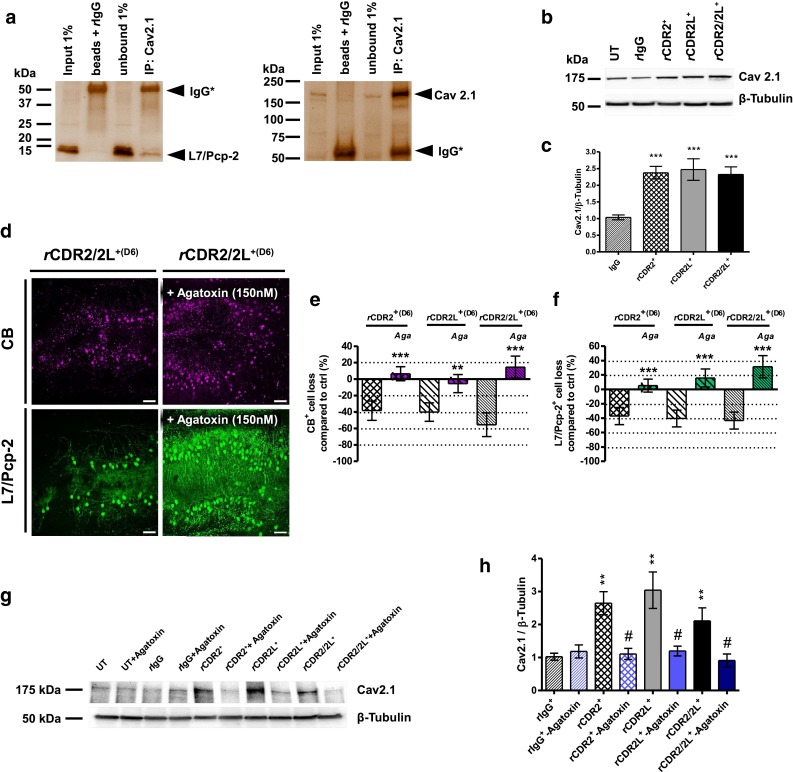
VGCC Cav2.1 expression is up-regulated by CDR-Ab internalization. **a** Rat cerebellar lysates were Co-IPed with anti-Cav2.1 and then detected by anti-L7/Pcp-2 in Western blot. Cav2.1 Co-IPed with L7/Pcp-2 (*left panel*) and IPed itself (control, *right panel*). *Arrowheads*: probed protein and nonspecific IgG* (heavy chain, 55 kDa). *Left*: molecular mass markers (kDa). Pathology of 125 ng/mL *r*CDR after 6 days correlated to VGCC. **b** Representative Western blot: Cav2.1 expression after *r*CDR treatment; **c** *r*CDR internalization induces twofold rise in Cav2.1 expression level (*n*_E_ = 19), **d** z-stack multiphoton micrographs: co-application of Cav2.1 antagonist ω-agatoxin (150 nM) prevents *r*CDR2/2L induced loss of CB^+^ (*magenta*) and L7/Pcp-2^+^ (*green*) PCs; *scale bars* 40 μm. Stereological counting of CB^+^ (**e**) and L7/Pcp-2^+^ (**f**) cells/mm^3^ supported the observed neuroprotective effect of ω-agatoxin during *r*CDR internalization for all tested groups (*n*_E_ = 7). **g** Representative Western blot: Cav2.1 expression after *r*CDR/ω-agatoxin co-treatment; **h** ω-agatoxin co-treatment blocks the *r*CDR-induced increase in Cav2.1 expression (*n*_E_ = 7). Data are mean ± SEM. Non-parametric two-tailed paired Mann–Whitney’s *U* test. *p < 0.05; **p < 0.01; ***p < 0.001; ^#^ *p* < 0.005; Table 1: CDR antibody effects in percentage

To confirm the suggested interaction of L7/Pcp-2 and Cav2.1, we performed Co-IPs on rat cerebellum extract and confirmed the protein complex (Fig. 5a). As a consequence of this interaction, the CDR antibody-induced loss of L7/Pcp-2 might influence the VGCC protein expression. To test whether VGCCs are affected by CDR internalization, we performed Western blot analysis of *r*CDR-treated cOTSC. We found that *r*CDR internalization increased the protein expression of Cav2.1 by more than twofold (100 ng/mL, 6 days, all groups compared to *r*IgG controls, ***p < 0.0001; Fig. 5b, c) and co-treatment with VGCC antagonist ω-agatoxin (150 nM) blocked this effect (Fig. 5g, h; ^#^ *p* < 0.005; *n*_E_ = 6). ω-agatoxin co-treatment averted CB^+^- and L7/Pcp-2^+^-PC loss (Fig. 5d–f). The rescue of CB and L7/Pcp-2 immunoreactivity was most strongly pronounced in *r*CDR2/2L^+^/ω-agatoxin co-treated cOTSC (***p < 0.0001, 6 days; *n*_E_ = 5; Fig. 5e, f; Table 1). ω-Agatoxin at 150 nM did not affect the CB^+^- and L7/Pcp-2^+^-PC count or Cav2.1 protein expression in *r*IgG and untreated naive controls.

**Fig. 6:**
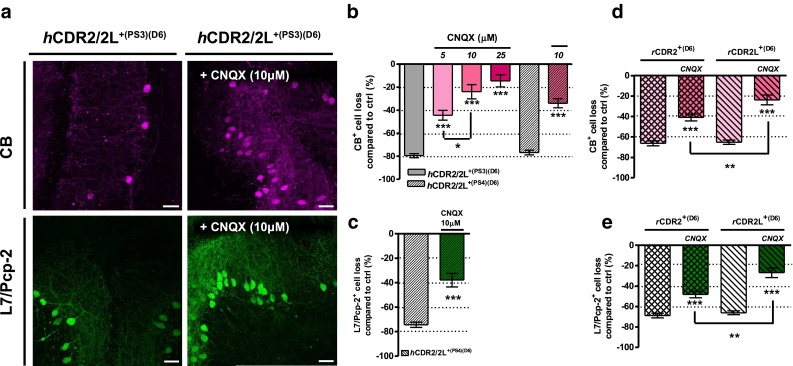
Indirect blockage of VGCC via AMPA receptor blockage attenuated *r*CDR-induced pathology. **a** z-stack multiphoton micrograph: indirect blockage of VGCC activity by blocking AMPAR activity with CNQX inhibits the observed *h*CDR2/2L^+(PS3)^-induced loss of CB (*magenta*) and L7/Pcp-2 (*green*) (*right* vs. *left panel*) (*h*CDR2/2L ^+(PS3)^: 4 μL/mL, CNQX: 10 μM; sample: day 6); *scale bars* 40 μm. Stereological counting of CB^+^ (**b**, **d**) and L7/Pcp-2^+^ (**c**, **e**) PCs showed that CNQX co-treatment reduced the *h*CDR2/2L- (4 μL/mL; CNQX: 5, 10, 25 μM; PS3: *n*_E_ = 6; PS4: *n*_E_ = 4) or *r*CDR-induced pathology (125 ng/mL, 10 μM, *n*_E_ = 6) in a concentration-dependent manner and more beneficially for *r*CDR2L than *r*CDR2 (CB: **p = 0.0071; L7/Pcp-2: **p = 0.0011). Data are mean ± SEM. Non-parametric two-tailed paired Mann–Whitney’s *U* test. *p < 0.05; **p < 0.01; ***p < 0.001; ^#^ *p* < 0.005; Table 1: CDR antibody effects in percentage

Inactivation of VGCC by blocking AMPA receptor-mediated synaptic depolarization with AMPA receptor antagonist CNQX revealed a similar rescue of CB^+^- and L7/Pcp-2^+^-PC loss (Fig. 6a–e; Table 1). We found that co-treatment of *h*CDR2/2L^+(PS3)^ (1 μL/mL) with CNQX minimized the CB^+^-PC loss significantly in a concentration-dependent manner (CNQX: 44 ± 8 % (5 μM), 24 ± 8 % (10 μM), 14 ± 6 % (25 μM); ***p < 0.0001, 6 days; *n*_E_ = 6; Fig. 6b). Similar CB^+^- and L7/Pcp-2^+^-PC loss reduction was obtained under 10 μM CNQX/*h*CDR2/2L^+(PS4)^ co-treatment (1 μL/mL *h*CDR2/2L^+(PS4)^; ***p < 0.0001; 6 days; *n*_E_ = 4; Fig. 6b, c; Table 1). The CDR-specific effects of CNQX (10 μM) were also tested with *r*CDR2 and *r*CDR2L (100 ng/mL, 6 days). *r*CDR/CNQX co-treatment partially rescued CB^+^-PCs (***p = 0.0001; *n*_E_ = 6; Fig. 6d; Table 1), with better rescue in *r*CDR2L/CNQX (63 %) than *r*CDR2/CNQX (38 %) (**p = 0.0054). The L7/Pcp-2^+^-PC count in co-treated *r*CDR2 or *r*CDR2L cOTSC with 10 μM CNQX showed reduction of L7/Pcp-2^+^-PC loss to 48 ± 3 % (*r*CDR2/CNQX) and 27 ± 5 % (*r*CDR2L/CNQX), respectively (***p < 0.0001; 6 days; *n*_E_ = 4; Fig. 6e; Table 1). The CNQX co-treatment was more beneficial for *r*CDR2L than for *r*CDR2, as seen with CB staining (L7/Pcp-2^+^-PC rescue: 30 % (CDR2) vs. 60 % (CDR2L), **p = 0.0011). The number of CB^+^- and L7/Pcp-2^+^-PCs in the *r*IgG control was not affected by the AMPA receptor antagonist.

Taken together, these data show that reducing Ca^2+^ influx through VGCC mitigates the CDR antibody-induced pathology by lowering intracellular Ca^2+^ levels.

### PKCγ is up-regulated by CDR2 and CDR2/2L antibodies

**Fig. 7:**
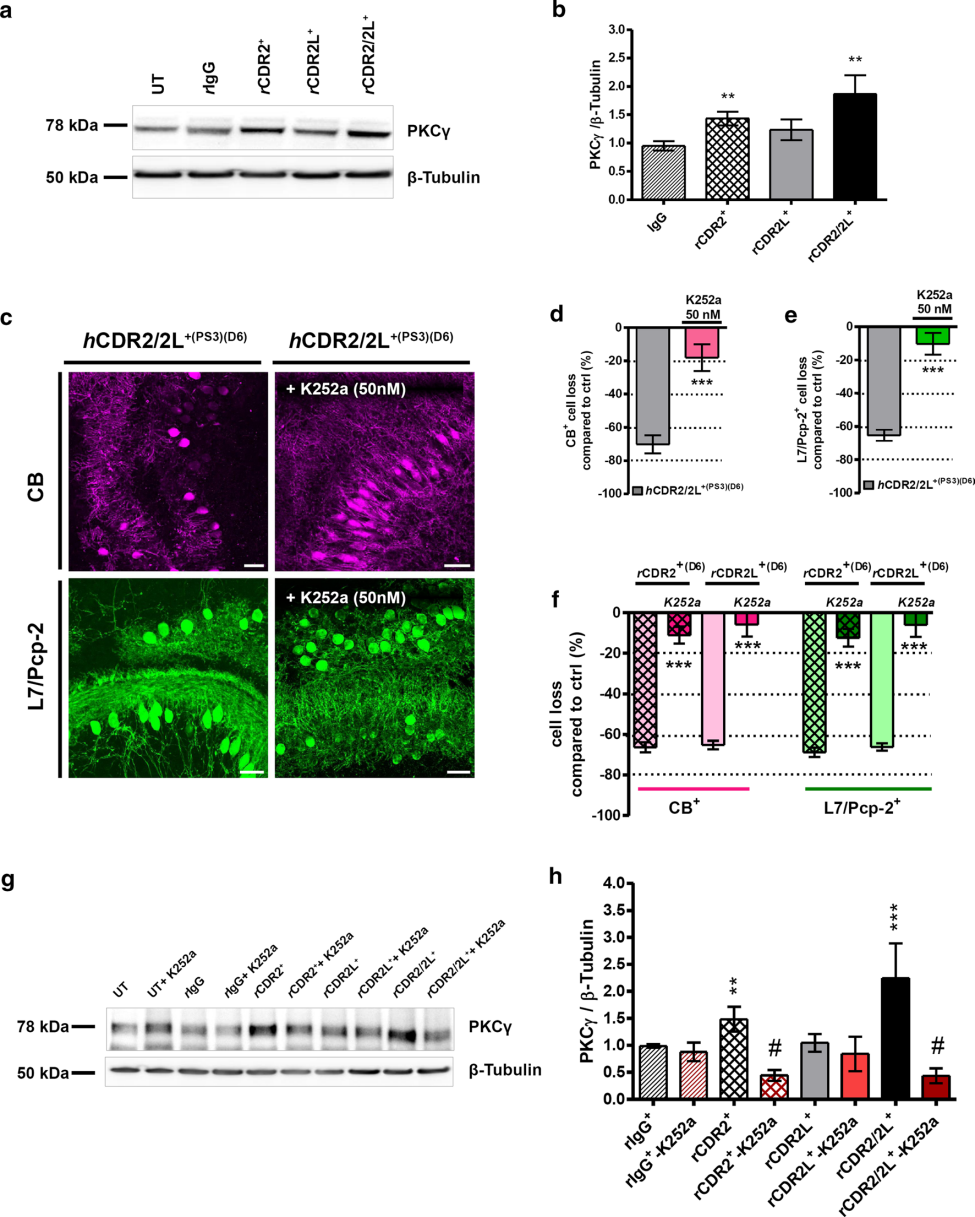
Protein kinase C gamma expression is up-regulated by CDR2 and CDR2/2L-Ab internalization. **a** Representative Western blot: PKCγ expression after *r*CDR internalization. **b** PKCγ expression was significantly increased after CDR2 and CDR2/2L internalization (125 ng/mL; 6 days; *n*_E_ = 14). **c** Multiphoton micrographs demonstrate that the *h*CDR2/2L^+(PS3)^-induced loss of CB (*magenta*) and L7/Pcp-2 (*green*) was minimized by PKCγ antagonist K252a (50 nM) co-treatment; *scale bars* 40 μm. Stereological counting of CB^+^ and L7/Pcp-2^+^ PCs in the obtained micrographs supported the positive effect of K252a on CDR antibody-induced pathology by showing a loss of <10 % compared to control (*h*CDR2/2L^+(PS3)^ [4 μL/mL] **d** CB: *n*_E_ = 4; **e** L7/Pcp-2: *n*_E_ = 4; **f** *r*CDR2 and *r*CDR2L [125 ng/mL] CB: *n*_E_ = 6; L7/Pcp-2: *n*_E_ = 6). **g** Representative Western blot: PKCγ expression after *r*CDR/K252a co-treatment. **h** K252a co-treatment reduces the *r*CDR-induced PKCγ expression rise in the *r*CDR2 and *r*CDR2/2L group (125 ng/mL; *n*_E_ = 5) after K252a co-treatment. Investigated samples: 6 days of CDR internalization; data in mean ± SEM; non-parametric two-tailed paired Mann–Whitney’s *U* test. *p < 0.05; **p < 0.01; ***p < 0.001; ^#^ *p* < 0.003; Table 1: CDR antibody effects in percentage

The postsynaptic Ca^2+^ transient can be modulated by inhibition of Ca^2+^-activated phospholipid-dependent protein kinase C (PKC) activity to prevent AMPA receptor-mediated synaptic depolarization [19, 73]. PKC is associated with apoptosis in cerebellar ischemia and stroke [8]. Missense mutations in PKCγ gene cause spinocerebellar ataxia type 14 [58, 60]. We found that 6 days of *r*CDR2 and *r*CDR2/2L internalization significantly increased PKCγ protein expression (*r*CDR2: 1.43 ± 0.12, **p = 0.0079; *r*CDR2/2L: 1.62 ± 0.29, *p = 0.0250; Fig. 7a, b), but not *r*CDR2L (1.23 ± 0.18, *p* = 0.1810; Fig. 7b). Therefore, we co-administrated 50 nM K252a to inhibit PKC activity during CDR internalizations. Blocking PKC activity with K252a prevented CDR antibody-induced CB^+^- or L7/Pcp-2^+^ -PC (*h*CDR2/2L^+(PS3)^[1 μL/mL]; 6 days; ***p < 0.0001; *n*_E_ = 4; and *r*CDR [125 ng/mL]; ***p < 0.0001; 6 days; *n*_E_ = 4; Fig. 7c–f; Table 1) and blocked the increased PKCγ protein expression caused by *r*CDR2 and *r*CDR2/2L internalization (Fig. 7g, h; ^#^ *p* < 0.003; *n*_E_ = 6). *r*CDR2L internalization had no effect on PKCγ protein expression, while CB^+^ - and L7/Pcp-2^+^-PC loss was rescued by K252a co-treatment in the *r*CDR2L group. The antagonist had no effect on the CB^+^- or L7/Pcp-2^+^ count or PKCγ protein expression in the *r*IgG and untreated naive control.

The results show that the CDR antibody-mediated pathology can be attenuated in the presence of VGCC, AMPA receptor, and PKC signaling inhibition.

### Ca^2+^-dependent protease calpain-2 is activated by CDR antibody internalization

**Fig. 8:**
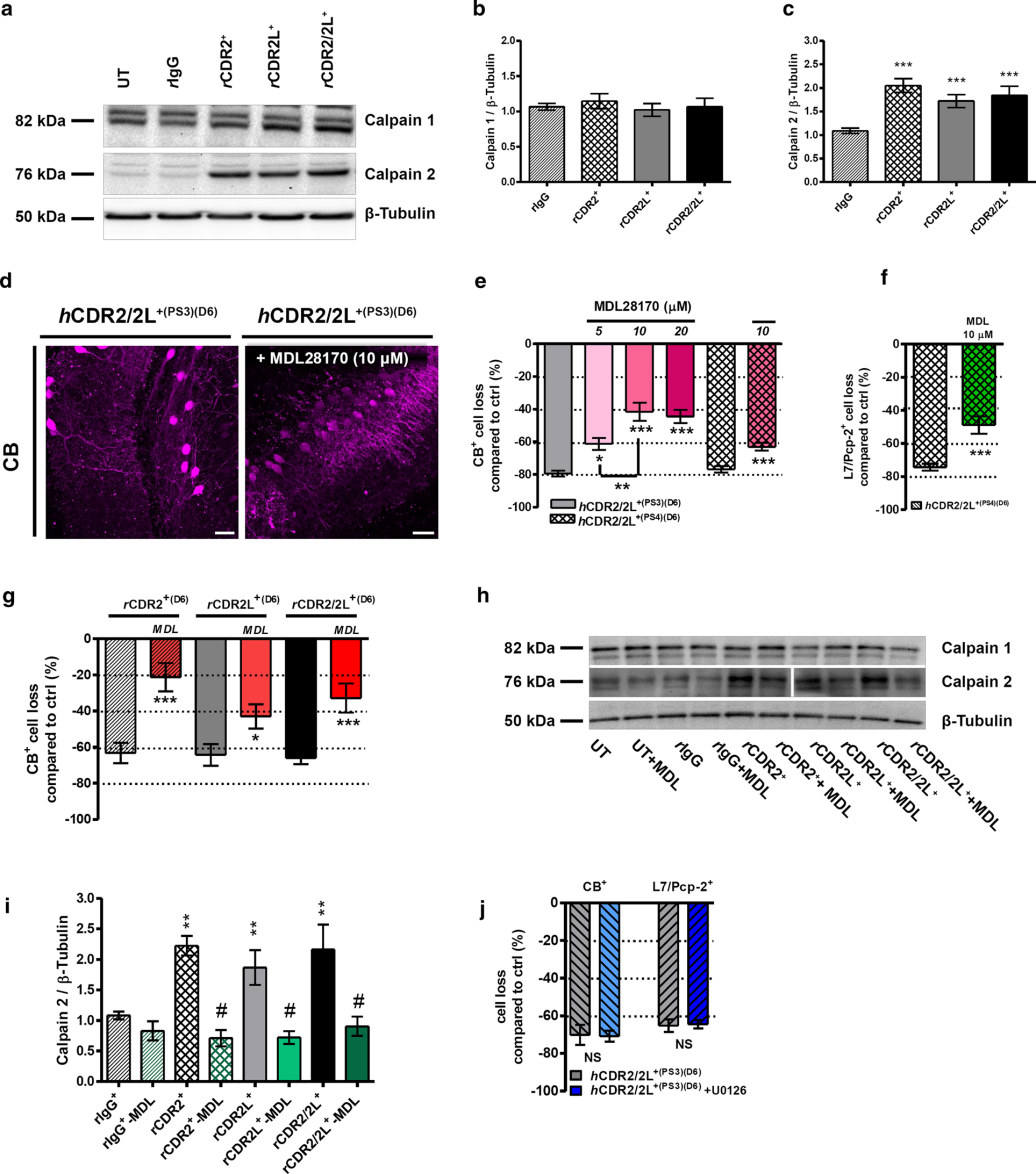
Calpain-2 activity is increased by CDR-Ab internalization, but not MAP kinase. **a** Representative Western blot: calpain-1 and calpain-2 expression after *r*CDR internalization and MDL28170 co-treatment (**h**). **b** *Bar plots* show that calpain-1 expression is not affected, but **c** calpain-2 expression is significantly increased after *r*CDR internalization (125 ng/mL; D6; *n*_E_ = 21) and can be **i** blocked by co-treatment with calpain antagonist MDL28170 (125 ng/mL + 10 μM MDL28170; *n*_E_ = 5). Calpain antagonist MDL28170 reduced the CDR-induced CB^+^ and L7/Pcp-2^+^ PC loss. **d** z-Stack multiphoton micrographs: co-treatment with calpain antagonist MDL28170 (10 μM) beneficially affects PC anti-CB (*magenta*) staining after *h*CDR2/2L^+(PS3)^ internalization (4 μL/mL, 6 days); *scale bars* 40 μm. Stereological counting of CB^+^ (**e**) and L7/Pcp-2^+^ (**f**) cells/mm^3^ in these micrographs showed that *h*CDR2/2L^+(PS3)^/MDL28170 treatment (5, 10, 20 μM) reduced the CDR-induced loss of CB [*n*_E_ = 6 (PS3) and *n*_E_ = 4 (PS4)] and L7/Pcp-2 [n_E_ = 3 (PS4)]. **g** Similar observation was found for *r*CDR/MDL28170 co-treatment (125 ng/mL, 10 μM MDL28170, CB: *n*_E_ = 5). Calpain antagonist reduced the CDR-induced loss depending on the CDR target to up to 60 % (Table 1). This was most pronounced for CDR2. **j** MAP kinase antagonist U0126 (5 μM) does not influence the loss of CB^+^ or L7/Pcp-2^+^ PCs after *h*CDR2/2L^+(PS3)^ internalization (1 μL/mL; 6 days; *n*_E_ = 4). Data are mean ± SEM. Non-parametric two-tailed paired Mann–Whitney’s *U* test. *p < 0.05; **p < 0.01; ***p < 0.001; ^#^ *p* < 0.008. Table 1: CDR antibody effects in percentage

Changes in VGCC, AMPA receptor, and PKC signaling can lead to elevated intracellular Ca^2+^ levels and induce Ca^2+^-dependent proteases activity. To examine whether CDR internalization affects Ca^2+^-dependent proteases activity of calpain-1 and calpain-2, we performed Western blot analysis of *r*CDR-treated cOTSC lysates. Calpain-1 expression was not affected, but calpain-2 expression was significantly increased (*r*CDR2: 2.05 ± 0.14, ***p < 0.0001; *r*CDR2L: 1.72 ± 0.14, ***p = 0.0002; *r*CDR2/2L: 1.84 ± 0.19, **p = 0.0012; 125 ng/mL; 6 days; Fig. 8a–c). We then investigated whether calpain-2 over-activation/-expression could be antagonized by calpain-specific inhibitor MDL28170, a known neuroprotective substrate in ischemia and traumatic injury models [9, 36]. MDL28170 co-application with *h*CDR2/2L^+(PS3/PS4)^ (6 days) reduced the CB^+^-PC loss to ~45 %, as well as the CDR-induced pathology on the dendritic arborizations in the remaining PCs (*n*_E_ = 6 for PS3 and *n*_E_ = 4 for PS4; Fig. 8d, e; Table 1). We found no difference between the 10 (42 ± 9 %) and 20 μM (44 ± 7 %) MDL28170/*h*CDR2/2L^+(PS3)^ treatment (*p* = 0.3841; Fig. 8e; Table 1). The MDL28170-induced rescue of CB^+^-PCs differed between *h*CDR2/2L^+(PS4)^ (18 %) and *h*CDR2/2L^+(PS3)^ (48 %) (*p = 0.0207; 10 μM; Fig. 8e; Table 1). Furthermore, *h*CDR2/2L^+(PS4)^-induced L7/Pcp-2^+^-PC loss was partially rescued by 10 μM MDL28170 (***p < 0.0001; *n*_E_ = 3; Fig. 8f; Table 1). MDL28170 (10 μM) co-treatment of *r*CDR2, *r*CDR2L, and *r*CDR2/2L-treated cOTSC (125 ng/mL, 6 days) showed an improvement of the dendritic morphology and significant reduction of CB^+^-PC loss (***p < 0.0001; *n*_E_ = 5; Fig. 8g; Table 1). The increase of calpain-2 expression seen during CDR internalization was blocked by 10 μM MDL28170 (Fig. 8h, i; ^#^ *p* < 0.008; *n*_E_ = 6).

These data show that CDR internalization leads to an increase in intracellular unbound Ca^2+^ to the millimolar range, which thereby activate the Ca^2+^-dependent protease calpain 2.

### MAP kinase signaling is not affected by CDR antibody internalization

Activation of mitogen-activated protein (MAP) kinase signaling cascades are associated with PKC-dependent synaptic depression/declustering of glutamate receptors, inflammation, death receptor activation, apoptosis, and oxidative stress [12, 23, 35]. Co-treatment of cOTSC with *h*CDR2/2L^+^ (1 μL/mL, 6 days) and MAP kinase inhibitor U0126 (5 μM) did not prevent the CB^+^- or L7/Pcp-2^+^-PC immunoreactivity loss (CB: *h*CDR2/2L^+(PS3)^: 70 ± 10 vs. 68 ± 7 % (5 μM U0126); *p* = 0.4268; L7/Pcp-2: *h*CDR2/2L^+(PS4)^: 65 ± 3 vs. 64 ± 2 % (5 μM U0126), *p* = 0.5249; *n*_E_ = 4; Fig. 8j).

These data suggest that the MAP kinase signaling cascade is not involved in the CB or L7/Pcp-2 immunoreactivity reduction induced by *h*CDR2/2L.

## Discussion

CDR antibodies, also known as onconeuronal Yo antibodies, have attracted increasing interest due to their suggested pathological role in the progressive loss of PCs seen in PCD after binding to CDR2 and CDR2L antigens [22, 31, 33, 70]. Because the mechanisms underlying PCD, in particular the effects of CDR antibodies, are largely unknown, we investigated whether they are internalized into PCs and how that affects PC physiology.

### Antibody-mediated PCD model: cerebellar organotypic slice culture (cOTSC)

OTSC can be used to study neurochemical, structural, and physiological changes linked to diseased in vivo brains [13, 44]. Hence, we created an ex vivo antibody-mediated PCD model by applying CDR-positive patients’ sera and affinity-purified rabbit CDRs to the culture medium of cerebellar rat OTSC. These slices offer unique advantages because the tissue architecture is preserved, synaptic circuitries are maintained, and various treatments can be evaluated without the influence of activated immune cells and the blood–brain barrier (BBB) [13, 21, 42–44]. The mechanisms of autoimmunity in paraneoplastic neurological diseases are complex and not yet understood. It is suggested that brain epitope-specific serum Abs can access the brain only if the integrity of the BBB is compromised [18, 20]. However, brain epitope-specific cerebrospinal fluid Abs can be produced by intrathecal synthesis due to activated T and B cells that can cross the BBB [51, 62]. Data from anti-Yo/CDR2-Ab positive PCD patients are contradictory in terms of cytotoxic T cell involvement [2, 3, 10, 56, 61, 65]. Therefore, it is of interest that PCs can internalize IgGs and that anti-Yo Abs can lead to cell injury and non-apoptotic death without the influence of activated immune cells [31, 33].

Here, we demonstrate that both *h*CDR and *r*CDR can cause similar PC pathology. By using *r*CDR we exclude cytotoxic T cell involvement, since the cOTSC was not exposed to related peptides which could activate brain-naive resident T cells [42]. Therefore, our data are most likely immune cell independent. We found that both CDR2 and CDR2L are internalized independently and their removal can partly reverse the calpain-2-dependent, but caspase-3-independent, pathology. We found equal effects of CDR2 and CDR2L on PC survival, although only a combination of CDR2/2L is associated with PCD [22]. However, CDR2/2L pathology was stronger, e.g., PKCγ results, and reciprocal Co-IP showed a CDR2–CDR2L complex in PCs, which supports the hypothesis that increased Ab avidity enhances the pathological effects [71]. Our CDR-induced pathology followed a similar time frame as described by Greenlee and colleagues [31], but the CDR internalization mechanisms remain to be explored. A recent study by Congdon et al. [14] demonstrated antibody internalization via IgG-FcγII receptor endocytosis. IgG-FcγII receptors are found in PC and regulate cerebellar function [47].

### CDR internalization affects postsynaptic signaling and thereby Ca^2+^ homeostasis: AMPAR–VGCC–PKC–calpain

**Fig. 9:**
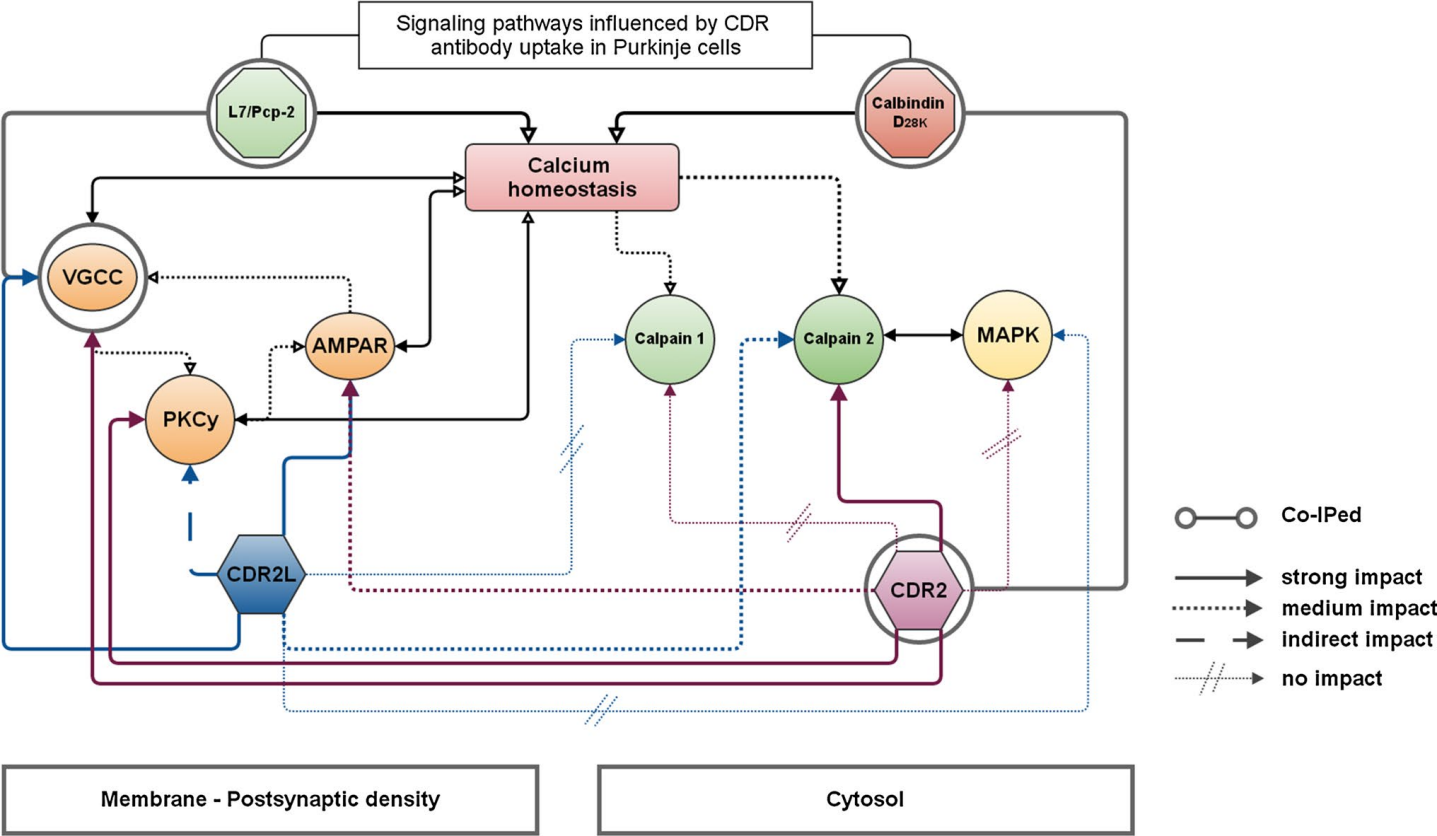
Cell Ca^2+^ homeostasis regulating signaling pathways influenced by CDR antibody internalization in Purkinje cells. The flowchart clarifies how the cell Ca^2+^ homeostasis is affected by CDR2 and CDR2L antibody internalization. In our CDR antibody-mediated PCD model, we found that (1) CDR2 interacts with calbindin D_28K_, (2) both CDR2 and CDR2L antibodies attenuate the Ca^2+^-buffering activity of calbindin D_28K_, (3) modify the VGCC modulator L7/Pcp-2, and (4) cause increase protein levels of VGCC and PKC. These events dramatically increase the levels of unbound cytoplasmic Ca^2+^ to the millimolar range and thereby induce over-activation of calpain-2. (5) It is known that calpain over-activation causes rapid modifications of synaptic membrane-associated proteins (VGCC, AMPAR), cytoskeleton proteins, major postsynaptic density scaffolding proteins, and synaptic protein kinases (PKC) and phosphatases. To prevent the enhanced Ca^2+^ influx through VGCC and free the CB binding capability by lowering the unbound cytoplasmic Ca^2+^ could be a putative neuroprotective treatment strategy. Antagonists to VGCC (ω-agatoxin), AMPA receptor (CNQX), PKCγ (K252a) and calpain (MDL28170) showed satisfactory neuroprotective impacts. Our data suggest that CDR2 are more likely interacting with cytosolic components and therefore responsible for calpain activation (K252a and MDL28170 treatment), whereas CDR2L are more likely to modify the postsynaptic density structure and physiology (ω-agatoxin and CNQX treatment). Therefore, both CDR antibodies can cause fatal alterations in the Purkinje cell signaling pathways and membrane structures by affecting actin remodeling, membrane reorganization, and protein trafficking, thereby cause neurodegeneration

In Fig. 9 we show the complexity, by which *h/r*CDR internalization changes PC physiology by targeting postsynaptic signaling factors and thereby modifies Ca^2+^ homeostasis. We described these factors along with related neurological diseases in Table S1. We found that the immunoreactivity of Ca^2+^ homeostasis regulator CB and VGCC modulator L7/Pcp-2 was reduced. These proteins are functionally linked, since CB shapes the post-tetanic potentiation induced by the Ca^2+^ influx through AMPAR and VGCC and can act as an activity-dependent sensor [32, 57]. When endogenous CB levels are reduced, PC dendritic outgrowth and differentiation is inhibited, and motor coordination and sensory integration fail [1, 4, 37, 74]. We found that the total CB protein levels are not affected by CDR internalization. However, Co-IP reveals that CDR2–CB forms a strong protein complex and therefore CDR2 antibody binding to CDR2 protein in the cell may disrupt CB function by sequestering CDR2 and thus hijacking CB’s interacting partner. “CDR antibody washout” restored the CB immunoreactivity only partially, indicating irreversible modifications of the CB Ca^2+^-binding mechanism or protein structure, as seen in other neurodegenerative diseases [11, 50].

CB dysfunction or deletion can result in drastic fast intracellular Ca^2+^-transient elevation which greatly influences Ca^2+^ homeostasis [4]. In PC signaling, intracellular Ca^2+^ microdomain variations due to AMPAR, VGCC, or PKC activity play a crucial role [24, 27, 34, 57, 68]. AMPAR activation by glutamate binding and site-specific PKC and receptor tyrosine kinases’ phosphorylation provides the depolarization necessary for opening VGCCs and therefore increases Ca^2+^ influx [19, 32, 57, 73]. We found that the P/Q-type VGCC modulator L7/Pcp-2 immunoreactivity is reduced and Cav2.1 (P/Q-type VGCC) protein concentration is increased by twofold after CDR internalization. L7/Pcp-2 modulates VGCC function in a concentration-dependent manner and enhances or dampens VGCC kinetics to shape fast Ca^2+^ influx in synaptic Ca^2+^ microdomains [39]. Inhibiting VGCC activity directly (ω-agatoxin) or indirectly (CNQX) prevented or reduced dramatically the CDR-induced CB and L7/Pcp-2 loss, respectively. CDR2L-induced pathology was more effectively reversed than CDR2 during AMPAR inhibition. Data from primary PC culture and synaptoneurosome preparation (data not shown) support that CDR2L is synaptic and could thereby be involved in membrane-associated signaling cascades. CB and L7/Pcp-2 dysfunction and VGCC up-regulation will promote excessive Ca^2+^ influx into PCs, which can activate cell death pathways by enhancing PKC and Ca^2+^-dependent protease activity [41].

PKC is associated with apoptosis in ischemia and stroke [8, 29, 60]. In PCs, enhanced PKC activity will reduce dendritic differentiation, whereas reduced PKC activity increases it [45, 46]. We found that the up-regulation of PKCγ expression under CDR internalization was accompanied by reduced dendritic arborizations and loss of tertiary branches. In retina, L7/Pcp-2 interacts with PKC [64] and we found that the increased PKC activity led to reduced L7/Pcp-2 expression (data not shown). Inhibition of PKCγ activity prevented the CDR-induced morphological changes, the loss of CB as well as L7/Pcp-2 immunoreactivity and blocked the increased PKCγ protein expression. However, the increase in PKCγ expression was only seen for CDR2 and CDR2/2L, but not for CDR2L internalization, although K252a rescued the CB and L7/Pcp-2 immunoreactivity in all *r*CDR-treated cOTSCs. We therefore hypothesize that CDR internalization enhances PKC activity, which: first, reduces L7/Pcp-2 expression; second, increases Cav2.1 expression; third, increases intracellular Ca^2+^, which activates Ca^2+^-dependent proteases such as calpain-1 and calpain-2 and thereby induces neuronal death.

Calpain-1 and calpain-2 are activated by different cellular Ca^2+^ concentrations. In Alzheimer’s, Huntington’s, and Parkinson’s disease, calpain over-activation or dysregulation triggers neuronal death by truncation of important synaptic substrates [5, 16, 17, 26, 53, 59]. Western blot analysis revealed that CDR internalization significantly increased calpain-2 protein expression, but not calpain-1 which indicates a intracellular Ca^2+^ level above 0.250 mM [75]. Inhibiting calpain activity by calpain antagonist MDL28170 reduced the CDR-induced CB loss and blocked calpain-2 protein enhancement. Interestingly, the CDR2L induced pathology is less affected by calpain inhibition than CDR2. This may be explained by the distribution of CDR2 (cytosol) and CDR2L (membrane) proteins [22]. We hypothesize that CDR internalization affects activity-dependent modifications of synaptic integrity, stability, and function of target proteins, which modulates the synaptic Ca^2+^ transient by mediating over-activation of calpain-2 [5, 17]. As calpain-mediated truncation of substrates is regulated by their phosphorylation state, there is a possibility of cross talk between calpain activation and activation of mitogen-activated protein (MAP) kinase. MAP kinase signaling cascades are associated with PKC-dependent synaptic depression and declustering of receptors, inflammation, death receptors’ activation, apoptosis, and oxidative stress [12, 23, 35]. Althought PKC inhibition prevented CDR-induced CB and L7/Pcp-2 loss, blockage of MAP kinase activity did not show any rescue effects.

## Conclusion

PCD pathogenesis is largely unknown. We propose a two-step pathology mechanism. First, internalization of CDR antibodies modifies important regulatory factors of Ca^2+^ homeostasis, which lead to the silencing of the PCs. Second, cytototoxic T cells and microglia mediate the clearing of diseased cells, as known from autopsy studies [61].

CDR antibodies play an important role in the PCD pathogenesis by inducing PC loss, which causes severe ataxia, dysarthria, diplopia, and vertigo in PCD patients. In line with previous results, we have shown that such cerebellar findings can be linked to imbalance in intracellular Ca^2+^ homeostasis due to alterations of CB, VGCC, or PKC [1, 27, 40, 58, 69]. Therefore, lowering the intracellular Ca^2+^ levels by inhibition, the VGCC–AMPAR–PKC signaling pathway (Fig. 9) during the progress of PCD will beneficially modulate Ca^2+^ homeostasis by stabilizing the Ca^2+^-binding capability of CB and prevent the induction of the critical calpain response cascade. The strong neuroprotective effect of antagonizing VGCC–AMPAR–PKC signaling pathway during CDR antibody internalization may therefore be of potential clinical relevance.

## Electronic supplementary material

Below is the link to the electronic supplementary material.

Fig. S1 *h*CDR and *r*CDR internalization induces no apoptosis. (a) There is no pathology seen in hematoxylin and eosin stained, paraffin-embedded ultra thin sections of cOTSC after 6 days of *h*CDR2/2L^+(PS3)^ serum treatment. Purkinje (PCL), molecular (MCL) and granule (GCL) cell layer, scale bars 50 μm. (b) Double immunostaining of CB (magenta) and cleaved “active” caspase-3 (green) in *r*IgG and *r*CDR2/2L-treated cOTSC after 6 days. Orthogonal view shows that cleaved caspase-3 positive apoptotic cells were scattered in the cerebellum in both *r*IgG control and *r*CDR2/2L treated group. All of these cells were small and labeling was not co-localized with CB^+^ PCs; scale bars 15 μm. Supplementary material 1 (TIFF 4816 kb)

Fig. S2 *h*CDR and *r*CDR internalization induces GoLoco domain protein L7/Pcp-2 immunoreactivity loss. (a) L7/Pcp-2 (green) positive PCs are reduced under *h*CDR2/2L serum from PCD patient 3 and 4 at day 6 (D6); scale bars 40 μm. (b) Internalization of *h*CDR2/2L^+(PS3)^ and *h*CDR2/2L^+(PS4)^ (1 μL/mL) reduced the L7/Pcp-2 positive PCs (L7/Pcp-2^+^) to two-third over time (samples taken: day 2 (D2), day 4 (D4), day 6 (D6); (*n*_E_ = 3)). (c) *r*CDR internalization caused similar reduction pattern of L7/Pcp-2 (green) positive PCs after 6 days as seen for *h*CDR in (a), scale bars 40 μm. (d) *r*CDR2, *r*CDR2L and *r*CDR2/2L internalization led to ~70 % L7/Pcp-2^+^ PC loss with no difference between the groups (*n*_E_ = 6). Data in mean ± SEM. Non-parametric two-tailed paired Mann–Whitney’s *U* test. *p < 0.05; **p < 0.01; ***p < 0.001. The percentage changes to the controls are summarized in Table 1. Supplementary material 2 (TIFF 5778 kb)

Supplementary material 3 (DOC 59 kb)

Supplementary material 4 (DOC 29 kb)

Supplementary material 5 (DOC 51 kb)

## Abbreviations

Abs: Antibodies
AMPA: α-Amino-3-hydroxy-5-methyl-4-isoxazolepropionic acid
Ca^2+^: Calcium ion
CB: Calcium-binding protein calbindin D_28K_
CDR: Cerebellar degeneration-related protein
cOTSC: Cerebellar organotypic slice culture
IgG: Immunoglobulin G
L7/Pcp-2: Purkinje cell-specific protein-2
PC: Purkinje cell
PCD: Paraneoplastic cerebellar degeneration
PKC: Protein kinase C
VGCC: Voltage-gated calcium channel
