# Supplementary figures and images for: Paraneoplastic CDR2 and CDR2L antibodies affect Purkinje cell calcium homeostasis
Source: Acta Neuropathol. 2014 Oct 24;128(6):835–52. doi: 10.1007/s00401-014-1351-6 (PMC4231287; doi:10.1007/s00401-014-1351-6)

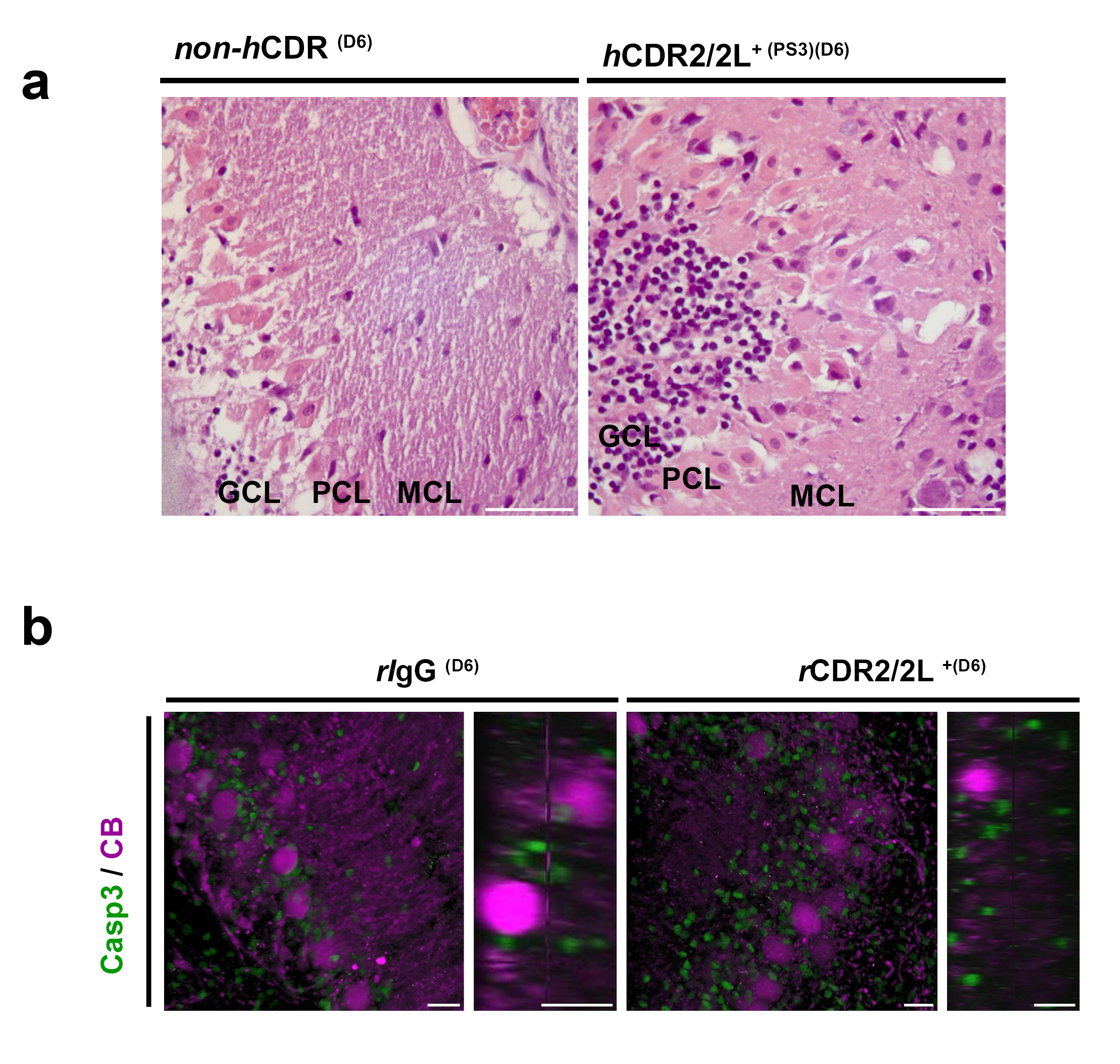

Supplement: Supplementary file 1 — hCDR and rCDR internalization induces no apoptosis. (a) There is no pathology seen in hematoxylin and eosin stained, paraffin-embedded ultra thin sections of cOTSC after 6 days of hCDR2/2L+(PS3) serum treatment. Purkinje (PCL), molecular (MCL) and granule (GCL) cell layer, scale bars 50 μm. (b) Double immunostaining of CB (magenta) and cleaved “active” caspase-3 (green) in rIgG and rCDR2/2L-treated cOTSC after 6 days. Orthogonal view shows that cleaved caspase-3 positive apoptotic cells were scattered in the cerebellum in both rIgG control and rCDR2/2L treated group. All of these cells were small and labeling was not co-localized with CB+ PCs; scale bars 15 μm. Supplementary material 1 (TIFF 4816 kb) [file 401_2014_1351_MOESM1_ESM.tif]

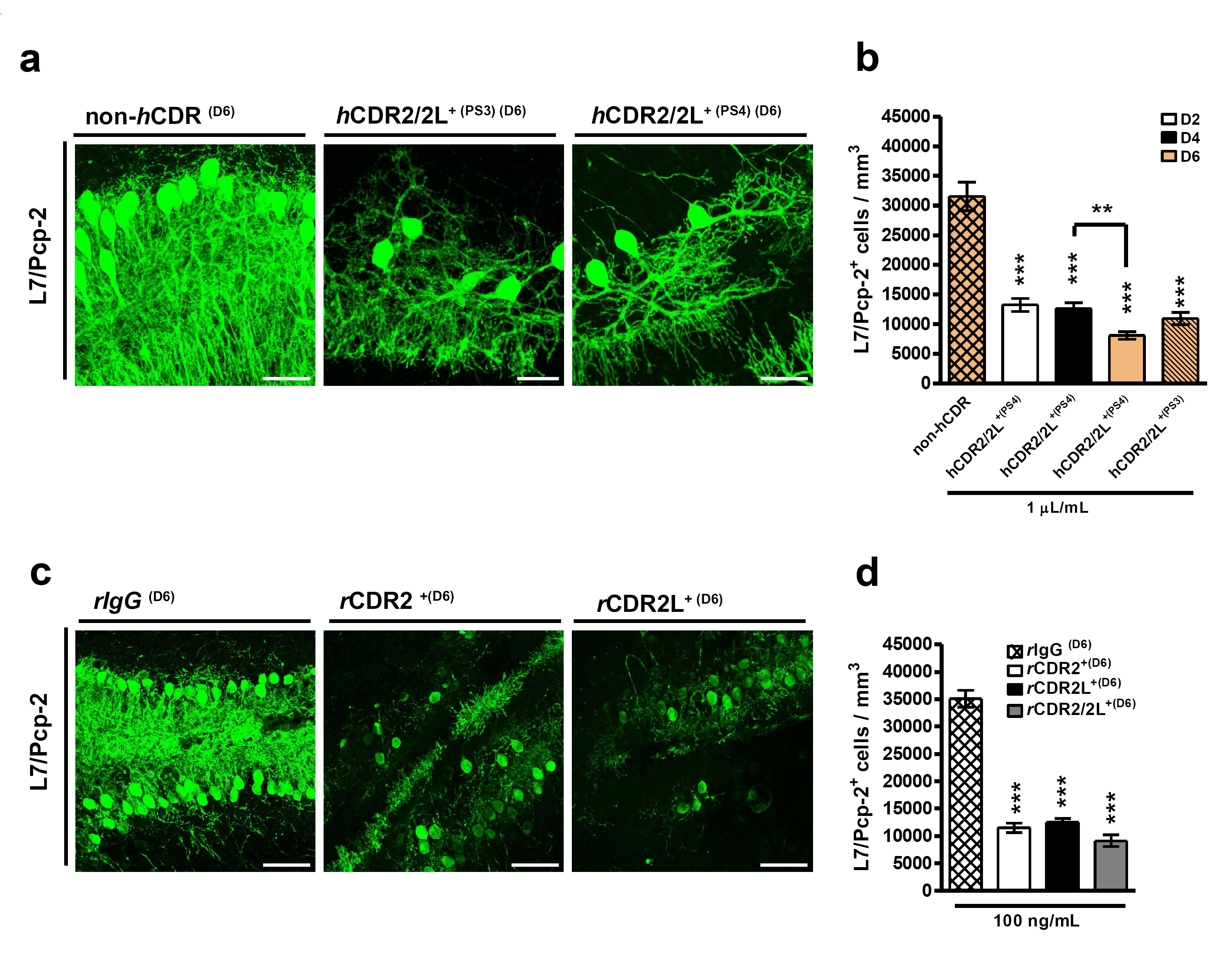

Supplement: Supplementary file 2 — hCDR and rCDR internalization induces GoLoco domain protein L7/Pcp-2 immunoreactivity loss. (a) L7/Pcp-2 (green) positive PCs are reduced under hCDR2/2L serum from PCD patient 3 and 4 at day 6 (D6); scale bars 40 μm. (b) Internalization of hCDR2/2L+(PS3) and hCDR2/2L+(PS4) (1 μL/mL) reduced the L7/Pcp-2 positive PCs (L7/Pcp-2+) to two-third over time (samples taken: day 2 (D2), day 4 (D4), day 6 (D6); (n E = 3)). (c) rCDR internalization caused similar reduction pattern of L7/Pcp-2 (green) positive PCs after 6 days as seen for hCDR in (a), scale bars 40 μm. (d) rCDR2, rCDR2L and rCDR2/2L internalization led to ~70 % L7/Pcp-2+ PC loss with no difference between the groups (n E = 6). Data in mean ± SEM. Non-parametric two-tailed paired Mann–Whitney’s U test. *p < 0.05; **p < 0.01; ***p < 0.001. The percentage changes to the controls are summarized in Table 1. Supplementary material 2 (TIFF 5778 kb) [file 401_2014_1351_MOESM2_ESM.tif]
