# Supplementary material for: Paraneoplastic CDR2 and CDR2L antibodies affect Purkinje cell calcium homeostasis
Source: Acta Neuropathol. 2014 Oct 24;128(6):835–52. doi: 10.1007/s00401-014-1351-6 (PMC4231287; doi:10.1007/s00401-014-1351-6)
Supplement: Supplementary file 3 — Supplementary material 3 (DOC 59 kb) [file 401_2014_1351_MOESM3_ESM.doc]

Table S1: Investigated factors including their functions and neurological implications

| **Factor** | **MW (kDa)** | **Function** | **Neurological implication** | **Reference** |
| --- | --- | --- | --- | --- |
| α-amino-3-hydroxy-5-methyl-4-isoxazolepropionic acid receptor [AMPAR] | 102-105 | - mediates the majority of fast excitatory transmissions in the CNS by depolarisation - implicated in synapse formation, stabilization, and plasticity - kinetic properties of AMPARs are fine-tuned by post-transcriptional and post-translational modifications | changes in the activity of AMPAR: Alzheimer’s, amyotrophic lateral sclerosis, stroke, epilepsy | 19, 32, 73, |
| Calpain 1;  Mu-type | 82 | - calcium-dependent cysteine protease - functionally active at normal (calpain 1; micromolar) and high (calpain 2;millimolar) calcium levels - regulate cellular processes by truncation of important synaptic substrates like cytoskeleton proteins, receptors (AMPAR), channels (VGCC), postsynaptic density proteins, protein kinases (MAPK) and by disrupting signal convergence between thyrosine kinase/phosphatase and calcium-mediated signal cascades - produce post-translational protein modifications | over-activation of calpain:  ischemia, stoke, epilepsy, traumatic nerve injury and neurodegenerative disorders like Alzheimer’s, Huntington’s and Parkinson’s | 5, 9, 16, 17, 26, 36, 41, 53, 59, 75, |
| Calpain-2;  M-type | 76 |
| Calbindin D28K [CB] | 28 | - Purkinje cell specific expressed marker of cell survival - high-affinity calcium binding protein (“EF-hand”); - modulates neuronal excitability by prolonging the decay time constant of fast AMPAR-mediated calcium transients | neuroprotective role, if reduced:  ataxia, neurodegeneration, impaired sensory integration | 1, 4, 6, 24, 28, 32, 37, 38, 57, 74 |
| Cerebellar degeneration-related antigen 2 [CDR2; anit-Yo]]  & 2like [CDR2L; HUMPPA] | 62  53 | - CDR2: interacts with c-myc, with cell cycle-related proteins and with protein kinase through its leucine zipper motif - CDR2: involved in signal transduction and gene transcription - CDR2Like: functions are unknown | anti-Yo mediated PCD | 15, 48, 49, 52, 54, 55, 66 |
| Purkinje cell specific protein 2 [L7/Pcp2] | 17 | - Purkinje cell specific expression - GoLoco domain protein which modulates the activation of Gα(i) and Gα(o) - differentially affects the Gα and Gβ,γ arms of receptor-induced G(i/o) signaling in a concentration-dependent manner, through which it increases the dynamic range of regulation of P/Q-type VGCC by G(i/o) protein-coupled receptors | not known | 39, 64 |
| Mitogen-activated protein kinase [MAPK] | 44  42 | - Serine/threonine protein kinases - involved in many cellular programs, such as neuronal differentiation, plasticity, motility, and may also modulate neuronal survival | over-activation of MAPK: inflammation, activation of death receptors, apoptosis, ischemia | 12, 23, 35, |
| Protein kinase C gamma [PKCγ] | 78 | - Serine/threonine protein kinase - activity is dependent on calcium and phospholipids - plays a key regulatory role in cell growth and differentiation, gene expression, hormone secretion and membrane function - PKC activity prevent AMPAR-mediated synaptic depolarization | over-activation can induce apoptosis: ischemia, stroke, neurodegeneration  miss-sense mutations of PKC:  spinocerebellar ataxia type 14 | 8, 19, 29, 41, 45, 46, 48, 58, 60, 64, 73 |
| Voltage-gated calcium channel [VGCC] | 235  175 | - involved in Ca^2+-^dependent processes (neurotransmitter release and gene expression) - mediates neuronal Ca^2+^ entry in response to membrane depolarization - Ca^2+^ currents are characterized on the basis of their biophysical and pharmacologic properties and include L-, N-, T-, P-, Q-, and R- types - P/Q-type Ca^2+^ channels like Cav2.1 are localized to presynaptic nerve terminals and their currents initiate a rapid synaptic transmission - P/Q-type channels play a prominent role at the inhibitory synapses afferent onto Purkinje cells | mutation in P/Q type VGCC:  spinocerebellar ataxia type 6, ataxia | 27, 40, 69 |
