# Supplementary material for: Paraneoplastic CDR2 and CDR2L antibodies affect Purkinje cell calcium homeostasis
Source: Acta Neuropathol. 2014 Oct 24;128(6):835–52. doi: 10.1007/s00401-014-1351-6 (PMC4231287; doi:10.1007/s00401-014-1351-6)
Supplement: Supplementary file 4 — Supplementary material 4 (DOC 29 kb) [file 401_2014_1351_MOESM4_ESM.doc]

Table S2: Patients

| **Number** | **Age (Years)** | **Sex** | **PNS** | **CDR antibodies** | **P/Q-VGCC antibodies** | **Cancer** |
| --- | --- | --- | --- | --- | --- | --- |
| PS1 | 75 | F | No | CDR2+ | No | Uterus |
| PS2 | 73 | F | No | CDR2L+ | No | Ovary |
| PS3 | 73 | F | **PCD** | CDR2/2L+ | No | Mamma |
| PS4 | 80 | F | **PCD** | CDR2/2L+ | No | Ovary |

F: Female; PCD: Paraneoplastic cerebellar degeneration; PNS: Paraneoplastic neurological syndrome; PS: patient serum; VGCC: Voltage-gated calcium channel
