# Supplementary material for: Paraneoplastic CDR2 and CDR2L antibodies affect Purkinje cell calcium homeostasis
Source: Acta Neuropathol. 2014 Oct 24;128(6):835–52. doi: 10.1007/s00401-014-1351-6 (PMC4231287; doi:10.1007/s00401-014-1351-6)
Supplement: Supplementary file 5 — Supplementary material 5 (DOC 51 kb) [file 401_2014_1351_MOESM5_ESM.doc]

Table S3: Used antibodies

| **Primary antibody** | **Source** | **Catalog No.** | **Host** | **Concentration** | | | | | | |
| --- | --- | --- | --- | --- | --- | --- | --- | --- | --- | --- |
|  |  |  |  | **Stock (μg/μL)** | **IHC** | | **WB** | | **IP (μg)** | **cOTSC (ng/mL)** |
| Calbindin D_28K_ | Sigma | C9848 | Mu | 5-7 | 1:2500 | | 1:2000 | | 2 |  |
| Calpain 1 | Abcam | AB28257 | Rb | 1 |  | | 1:1000 | |  |  |
| Calpain 2 | Abcam | AB39165 | Rb | 1 |  | | 1:1000 | |  |  |
| CDR2 | Sigma | HPA023870 | Rb | 0.2 |  | |  | |  | 20-400 |
|  | Proteintech | 11611-2-AP | Rb | 0.4 |  | |  | | 2 |  |
| CDR2Like | Sigma | HPA022015 | Rb | 0.1 |  | |  | | 2 | 20-400 |
|  | Proteintech | 14563-1-AP | Rb | 0.2 |  | |  | | 2 |  |
|  | Aviva Sys. Bio. | ARP39113 | Rb | 0.5 |  | |  | | 2 |  |
| Caspase 3  cleaved | Abcam | Ab52293 | Rb | 1 | 1:250 | |  | |  |  |
| Cav2.1 | Alomone | ACC-001 | Rb |  |  | | 1:1000 | | 2 |  |
| L7/Pcp-2 | Takara Bio | M194 | Rb | 2 | 1:1000 | |  | |  |  |
| PKCγ | Invitrogen-Novex | 133800 | Mu | 0.5 |  | | 1:2000 | |  |  |
| β-Tubulin | Sigma | T4026 | Mu |  |  | 1:500 | |  | |  |
